# Parthenolide ameliorated gout-like inflammation by inhibiting activation of NLRP3 inflammasome

**DOI:** 10.1016/j.chmed.2026.06.016

**Published:** 2026-06-30

**Authors:** Yonghong Cheng, Yinjing Jiang, Hui Wang, Haoqing Zhu, Yanling Wu, Jixing Nan, Lihua Lian

**Affiliations:** a Key Laboratory of Traditional Chinese Korean Medicine Research of State Ethnic Affairs Commission, College of Pharmacy, Yanbian University, Yanji 133002, China; b Key Laboratory of Natural Medicines of the Changbai Mountain, Ministry of Education, College of Pharmacy, Yanbian University, Yanji 133002, China

**Keywords:** gout, hyperuricemia, inflammation, NLRPS, parthenolide, phagocytosis

## Abstract

**Objective:**

Crystals of monosodium urate (MSU) are the cause of gout, a common inflammatory disease. Parthenolide (Par), sesquiterpene lactone, derived from feverfew (*Pyrethrum parthenium*), possesses potent analgesic, anti-inflammatory, and anticancer properties. However, it remains elusive whether and how Par modulates gout. This research was designed to explore the therapeutic effect and mechanism of Par in acute gout.

**Methods:**

Chondrocytes from rat knee joint and mouse peritoneal macrophages (MPMs) were stimulated by lipopolysaccharide (LPS) plus MSU. Combination of yeast extract (YE) with oxygen oxyazine (OXO) was administered to mice to simulate hyperuricemia. MSU crystals were injected into the mice’s paws or subcutaneously into the mice’s backs to establish acute gouty models.

**Results:**

Par blocked the activation of NOD-Like Receptor Pyrin Domains 3 (NLRP3) inflammasome by reducing the expression and release of inflammatory mediators in gouty mice and LPS plus MSU-stimulated MPMs, LPS plus MSU-stimulated chondrocytes as well. Par also promoted phagocytosis abilities of MPMs and chondrocytes to reduce inflammation. In addition, Par dose-dependently reduced the serum levels of uric acid (UA), xanthine oxidase (XOD), creatinine (CRE), and blood urea nitrogen (BUN) and ameliorated renal injury in mice with hyperuricemia.

**Conclusion:**

Par ameliorated uric acid metabolism disorders of hyperuricemia, and improved acute gout-like inflammation through the inhibition of NLRP3 inflammasome activation, indicating that Par may be a promising candidate for gout-targeted therapy.

## 1 Introduction

Gout is a common inflammatory arthritis disease caused by the deposition of monosodium urate (MSU) crystals with symptoms, like swollen, erythematous, feverish joints, and severe pain, suffering 41 million people worldwide (Danve, Sehra, & Neogi, 2021). Many factors contribute to the occurrence of gout, such as high purine diet, genetics, age, gender, etc (Major et al., 2018, Zhang et al., 2023). Among them, genetic factors and obesity are the main causes (Afinogenova, Danve, & Neogi, 2022). Gout typically progresses through four distinct phases: early-stage hyperuricemia, MSU crystal deposition, acute inflammation due to MSU crystal deposition, and late-stage gout worsening (Ragab, Elshahaly, & Bardin, 2017).

Hyperuricemia is a chronic metabolic disease caused by purine metabolism disorders, resulting in elevated levels of uric acid, which is a critical stage of gout (Copur et al., 2022, Zhang et al., 2022). Gout often develops hyperuricemia as a complication (Dalbeth, Gosling, Gaffo, & Abhishek, 2021). However, hyperuricemia will invariably develop gout, and nor does gout inevitably accompany hyperuricemia (Zhang, 2021). It is reported that approximately one-fourth of patients with hyperuricemia develop gout (Nian, & You, 2022). Uric acid salts, being metabolites, possess the capacity to innate immune memory, and the perturbation of uric acid metabolism in hyperuricemia, along with recurrent gout flares will argument non-specific immune responses, exacerbate inflammation, and precipitate damage to tissues and organs (Cabău, Crișan, Klück, Popp, & Joosten, 2020). Due to the close relationship between gout and hyperuricemia, it is undoubtedly an efficient approach for gout treatment to reduce uric acid levels while treating inflammation caused by MSU deposition.

The excessive deposition of MSU in the joint cavity exceeds the phagocytic and digestive capacity of innate immune cells (phagocytic cells), leading to cell death and triggering an inflammatory storm (Gordon, 2016). Specifically, the surface of MSU with uniformly negative charges is highly favored by A-class and B-class scavenger receptors on macrophages (Nakayama, 2018). MSU crystals engulfed by phagocytic cells will disrupt lysosomal membranes, resulting in the leakage of lysosomal contents, such as histone proteases (Cathepsin B) or adenosine triphosphate (ATP) leakage into the cytoplasm, causing potential cellular damage (Nakayama, 2018). Upon the engagement of cytoplasmic histone proteases and NOD-like receptor pyrin domains 3 (NLRP3), the activation of the inflammasome is initiated, subsequently leading to the active interleukin-1*β* (IL-1*β*) production. In turn, released IL-1*β* triggers a local inflammatory response in the joint cavity as well as the surrounding connective tissue (Gu et al., 2021).

The activation of NLRP3 inflammasome elicited by MSU crystals, concomitant with the ensuring release of inflammatory cytokine IL-1*β*, play a major role in gout flares (Nicola, Anna, Angelo, & Abhishek, 2021). There are two signals associated with NLRP3 activation and cell death. The first signal is that MSU induces pro-IL-1*β* and NLRP3 through the nuclear factor-*κ*B (NF-*κ*B) signaling pathway (Nakayama, 2018). Metabolic alterations or lipopolysaccharide (LPS) secretion, the accumulation of free fatty acids (FFAs), including palmitic acid, can activate the Toll-like receptor 2 (TLR2) or TLR4 signaling pathway, which in turn leads to the accumulation of pro-IL-1*β* within cells (Perez-Ruiz, & Dalbeth, 2019). The second signal is that MSU causes lysosomal damage and stimulates the assembly of complexes of various proteins [NLRP3, apoptosis-associated speck-like protein containing a CARD (ASC), and pro-Caspase-1], leading to the formation of inflammasomes. Active Caspase-1 processes pro-IL-1*β* into mature IL-1*β*, which is then released into the extracellular environment through damaged membranes of dying macrophages (Fu, & Wu, 2023).

Parthenolide (Par) (Fig. 1A), a sesquiterpene lactone obtained from *Pyrethrum parthenium* (L.) Sm.’s delicate leaves, shows several activities like anti-oxidant and anti-inflammatory effects (Wang et al., 2020). Par has been demonstrated to act as an inhibitor targeting NLRP3 inflammasome activation in high-fat diet-induced mouse hepatic steatosis, thereby ameliorating neurological impairments and neuroinflammation in animals with traumatic brain injury (Chinta et al., 2022, Ding et al., 2022). Nevertheless, the prospective impact of Par in gouty arthritis remains elusive. Therefore, this study investigated the potential effect and underlying molecular mechanism of Par in gouty arthritis.

**Fig. 1:**
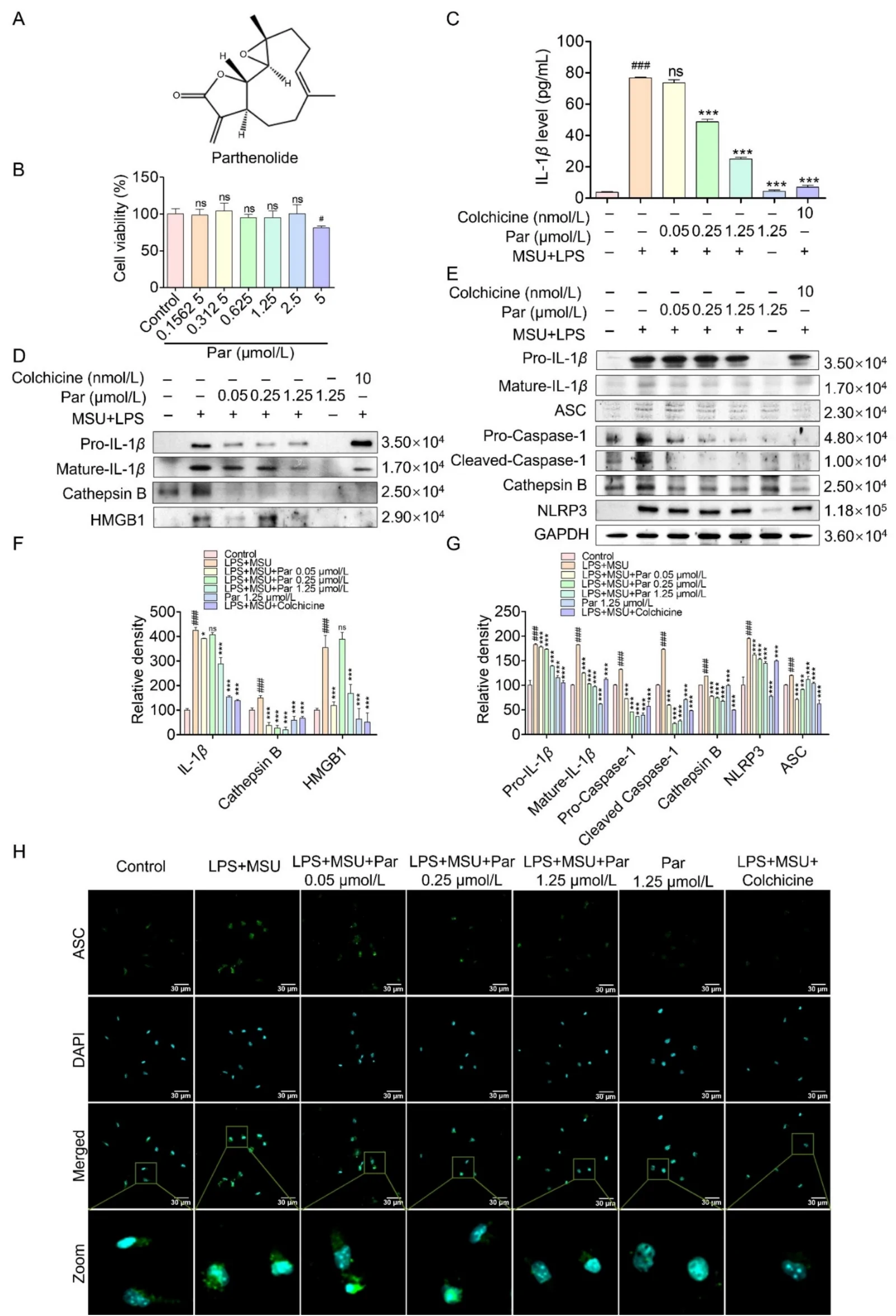
Par attenuated proinflammatory mediators in macrophages stimulated by MSU plus LPS. (A) Chemical structure of Par. (B) Cell viability of MPMs was determined by MTT assay. (C) Expression of IL-1*β* in MPMs supernatants determined by ELISA assay. (D) Expression of IL-1*β*, Cathepsin B, and HMGB1 in cell supernatants by Western blotting. (E) Expression of NLRP3, ASC, Caspase-1, Cathepsin B, and IL-1*β* in MPMs detected by Western blotting. (F) Relative density of the immunoreaction band from D. (G) Each immunoreaction band in cells is normalized for GAPDH. (H) Immunofluorescence staining of ASC (× 600, scale bar 30 μm). Mean ± SD, *n* = 3. ^#^*P* < 0.05, ^###^*P* < 0.001 *vs* control group; *P < 0.05, ^***^P < 0.001 *vs* LPS + MSU group; ns indicates no significant difference. DAPI: 4′,6-diamidino-2-phenylindole; ASC: apoptosis-associated speck-like protein containing a Caspase Recruitment Domain (CARD); HMGB1: high mobility group box-1; LPS: lipopolysaccharide; NLRP3: NOD-like receptor pyrin domains 3; CCK-8: cell counting kit-8; GAPDH: glyceraldehyde-3-phosphate dehydrogenase; MSU: monosodium urate.

## 2 Materials and methods

### 2.1 Materials

Par (PubChem CID: 7251185, purity ≥ 98.00%) and allopurinol (PubChem CID: 135401907, purity ≥ 98.00%) were purchased from Derike Bio-technology Co., Ltd. (Chengdu, China). Colchicine (Col, PubChem CID: 6167, purity ≥ 98.00%) was purchased from Push Bio-technology (Chengdu, China). Anti-F4/80 (ab6640), anti-NLRP3 (ab263899), anti-high mobility group box-1 (HMGB1, ab79823), anti-glyceraldehyde-3-phosphate dehydrogenase (GAPDH, ab8254), and anti-neutrophil elastase (NE, ab68672) antibodies were purchased from Abcam (Cambridge, MA, USA). Anti-Caspase-1 (14F468, sc-56036), anti-Cathepsin B (H-5, sc-365558), anti-IL-1*β* (11E5, sc-52012) and anti-ASC (sc-514414) antibodies were purchased in Santa Cruz Biotechnology, Inc. (Santa Cruz, CA, USA). Anti-IL-1*β* (AF401NA) antibody was purchased from R&D Systems (Minneapolis, MN, USA). Fetal bovine serum (FBS) and Dulbecco’s modified eagle’s medium (DMEM) were purchased from Baidi Biotech (Hangzhou, China). Enzymatic colorimetric kits were purchased from Nanjing Jian Cheng Biotechnology Research Institute (Nanjing, China). Methyl thiazolyl tetrazolium (MTT, M8180) and dimethyl sulfoxide (D8371) were purchased from Solarbio (Beijing, China). HRP Goat Anti-Rabbit IgG Antibody (K1223) and HRP Goat Anti-Mouse IgG Antibody (00001–1) were purchased from APExBIO technology (Texas, USA). The secondary antibody of anti-Caspase-1, anti-Cathepsin B, anti-F4/80, anti-IL-1*β* (11E5, sc-52012) and anti-ASC was HRP Goat Anti-Mouse IgG Antibody. The secondaryantibody of the rest other primary antibodies was HRP Goat Anti-Rabbit IgG Antibody.

### 2.2 Animal experimentation protocol

Male Kunming mice (8 − 10 weeks, 20 − 22 g) were purchased from the laboratory Animal Center of Yanbian University (SCXK(JI)2022–0005). Each experiment was conducted strictly according to guidelines of the Animal Care Committee of Yanbian University and authorized by the Animal Research Committee of Yanbian University (YD20230911003).

(1) After mice were acclimatized for 5 d, establishment of the hyperuricemia model (Li et al., 2022, Zhang et al., 2015), mice were randomly divided into seven groups (five mice per group): Control group, oxygen oxyazine (OXO) + yeast extract (YE) group, OXO + YE + Par groups (1, 2 and 4 mg/kg), Par group (4 mg/kg) and OXO + YE + allopurinol group (7 mg/kg). The control group and the Par group were given equal volumes of saline, and 6 h later, YE paste was administered to the remaining groups for a week. And on 4 − 7 d, the mice were treated with Par and allopurinol by gavage, and 1 h later, OXO was administered via intraperitoneal injection, while the Par group received Par by gavage without OXO and YE. Serum, kidneys, and livers were obtained from the mice when they were sacrificed and used for follow-up experiments (including biochemical assays and H&E staining). (2) After mice were acclimatized for 5 d, the establishment of the gouty arthritis model (Wu et al., 2019, Zhang et al., 2021), mice were randomly divided into seven groups (five mice per group): Control group, MSU group, MSU + Par groups (1, 2, and 4 mg/kg), Par (4 mg/kg) group and MSU + colchicine group (3 mg/kg). After 1 h gavage treatment with Par and colchicine (equal volume of saline was given to control group by gavage), and then 0.1 mL MSU crystals (40 mg/mL) were injected into mice paws, while paws of control group and Par group were injected with equal volumes of phosphate-buffered saline (PBS). Mice were sacrificed after 24 h, and the mice’s left paws were fixed in a neutral formalin solution after they were skinned, and then transferred to 0.1 mmol/L ethylenediaminetetraacetic acid disodium salt (EDTA) and used for follow-up experiments (including H&E staining and immunohistochemistry staining. The mice’s right paws were used for Western blotting. (3) After mice were acclimatized for 5 d, establishment of air pouch gout model (Bai et al., 2023, Huang et al., 2023), mice were randomly divided into seven groups (five mice per group): Control group, MSU group, MSU + Par groups (1, 2, and 4 mg/kg), Par (4 mg/kg) group and MSU + colchicine group (3 mg/kg). On the first day, the mice’s back skin was injected with 5 mL of sterile air. On the second day, additional air was added. The mice received gavage treatments of Par and colchicine on the third day (equal volume of saline was given to control group by gavage). Following 1 h, mice’s air pouches were injected with 1 mL of MSU (3 mg/mL), while air pouches of control group and Par group were injected with equal volumes of PBS for 6 h. Following the mice’s sacrifice, the skin on the back was collected and fixed in a neutral formalin solution and then used for the follow-up experiments: Western blotting, immunohistochemistry staining, H&E staining, and immunofluorescence staining.

### 2.3 Isolation and culture of mouse peritoneal macrophages (MPMs)

Male Kunming mice aged 8 − 10 weeks, 20 − 22 g, were intraperitoneally injected with 1 mL of 3.8% sterile thioglycolate medium (BD Biosciences, San Jose, CA, USA). After three days, mice were administered euthanasia and exposed the peritoneum by cutting open the epidermis. 5 mL of prechilled PBS containing 3% fetal bovine serum (FBS) was injected intraperitoneally into mice for lavage, and then the lavage fluid was collected and centrifuged at 4 °C and 300 × *g*. After discarding the supernatant, the Dulbecco’s modified eagle’s medium (DMEM) mixture containing 10% FBS was well blown up and incubated in an incubator. Macrophages in good condition were pretreated with 10 nmol/L colchicine and the safe dose (0.05, 0.25, and 1.25 μmol/L) of Par for 1 h (Thyberg, Piasek, & Moskalewski, 1980). LPS (1 μg/mL) plus MSU (500 μg/mL) were added to stimulate MPMs (Hwang, Yang, Park, & Kim, 2015).

### 2.4 Isolation and culture of rat articular chondrocytes

Male SD rats aged 2 weeks old, 20 − 30 g, were used to isolate chondrocytes. The cartilage tissue was isolated from the joint surface and cut into 1 mm^3^ pieces in sterile PBS. The pieces were then centrifuged at 4 °C and 1 000 × *g*, separated from the supernatant, and digested in 0.2% type II collagenase in DMEM overnight. After the cartilage fragments were filtered through 70 and 40 μm strainer twice and then centrifuged. The chondrocytes were resuspended in DMEM media after the supernatant was discarded. The following investigations employed chondrocytes from the 3rd to 5th generation*.* Chondrocytes in good condition were pretreated with 10 nmol/L colchicine and the safe dose of Par (0.05, 0.25, and 1.25 μmol/L) for 1 h (Thyberg, Piasek, & Moskalewski, 1980). LPS (1 μg/mL) plus MSU (200 μg/mL) were added to stimulate chondrocytes (Liu-Bryan, Pritzker, Firestein, & Terkeltaub, 2005).

### 2.5 Preparation of MSU crystals

Uric acid (800 mg), NaOH (1 mol/L), and double distilled water (DDW, 155 mL) were heated and stirred at 110 − 115 °C until fully dissolved and then cooled to reach room temperature. Using 1 mol/L HCl, the pH was raised to 7.0 until trace crystals started to form, centrifuged, and let stand overnight at 4 °C.

### 2.6 Cell viability assay

The MPMs were cultured in 96 well plates (1 × 10^4^ per well). After the cells adhered, Pars (0.156 25, 0.312 5, 0.625, 1.25, 2.5, 5 μmol/L) (Park, Kang, Hwang, Oh, & Byun, 2020) were treated to the cells for 24 h. MTT solution (0.5 mg/mL) was added for 3 h and then removed to add dimethyl sulfoxide (200 μL per well) for 10 min. Using the microplate reader (BioTek, Vermont, USA) to detect the content of purple formazan at 540 nm.

The chondrocytes were cultured in 96 well plates (1 × 10^4^ per well). After the cells adhered, Pars (0.156 25, 0.312 5, 0.625, 1.25, 2.5, 5 μmol/L) (Park, Kang, Hwang, Oh, & Byun, 2020) were treated to the cells for 24 h. Then adding CCK-8 solution (10 μL per well) for 4 h and detecting the optical density value by the microplate reader at 450 nm.

### 2.7 Biochemical assays

The blood collected from mice was centrifuged, and the serum was stored at − 80 °C. As directed by the kit, the serum concentrations of uric acid (UA), xanthine oxidase (XOD), blood urea nitrogen (BUN), and creatinine (CRE) were determined by enzymatic colorimetric kits (Nanjing Jiancheng Bioengineering Institute, China). For UA measurement, serum samples were diluted 10-fold at 50 °C, mixed with buffer solutions, and centrifuged at 3 000 × *g* for 5 min, and the absorbance was read at 690  nm. XOD activity was assayed using the xanthine oxidase assay kit (A002-1–1, Nanjing Jiancheng Bioengineering Institute, China) with absorbance measured at 530  nm. CRE and BUN levels were determined by colorimetric methods using the creatinine assay kit (C011-2–1, Nanjing Jiancheng Bioengineering Institute, China) and urea nitrogen assay kit (C013-2, Nanjing Jiancheng Bioengineering Institute, China), respectively, with absorbances measured at 546  nm and 640  nm. All samples were analyzed in triplicate, and concentrations were calculated from standard curves.

### 2.8 Western blotting

Twenty mg of mouse paw and skin tissues and 300 μL of lysis buffer (Solarbio, Beijing, China) were added into the tube. Lysis buffer (50 μL) was added for a well of 1 × 10^6^ cells (MPMs and chondrocytes). Then, the protein tubes were placed in the tissue breaker (Bullet Blender, Beijing, China) for 20 min of lysis and then centrifuged at 15 000 relative centrifugal force (RCF) and 4 °C for 15 min. The supernatant was collected and stored at − 80 °C for Western blotting detection. The BCA protein assay kit was used to measure the amount of protein. Protein was separated using sodium dodecyl sulfate–polyacrylamide gel (SDS-PAGE) and then placed on a PVDF membrane in the same amount. After the blocking of skim milk, the membrane was incubated with the applicable primary antibodies (1:1 000) and secondary antibodies (1:8 000). The target protein was observed using Tanon^TM^ High-sig ECL Western blotting substrate, and the density was measured using Image J software.

### 2.9 Enzyme‑linked immunosorbent assay (ELISA)

IL-1*β* concentration in MPMs supernatant was measured using the mouse IL-1*β* ELISA assay kit (BMS6002-2TEN, Invitrogen, Minneapolis, USA) as instructed. Specifically, the microwell strips were washed by washing buffer (100 μL per well) and added with standards (0, 31.25, 62.5, 125, 250, 500 pg/mL) and samples (50 μL per well) sequentially. And then biotin-conjugate (50 μL per well) was added and incubated for 2 h. After washing, streptavidin-HRP (100 μL per well) was added and incubated for 1 h. Following another washing, substrate solution (100 μL per well) was added for 10 min. At last, adding stop solution (100 μL per well) and detecting the optical density value by the microplate reader at 450 nm. Sample concentrations were calculated from a standard curve established using serial dilutions of the standards.

### 2.10 Hematoxylin and eosin (HE) and immunohistochemistry (IHC)

Paw and skin tissues were embedded and fixed in paraffin and frozen solution, respectively, and then cut into 5 μm tissue sections. H&E staining solution was then incubated to the tissue slices after they had been dried using ethanol gradients at varying concentrations. Tissue sections were soaked in sodium citrate buffer for 18 h to remove antigens following ethanol dewaxing. Endocannbiotin/affinity blocking kit, 5% goat serum, and 3% hydrogen peroxide solution were then used to block the tissue sections. The appropriate primary (anti-Ly6G, anti-F4/80, and anti-NLRP3) and secondary (enzyme-labeled goat anti-rabbit/mouse IgG polymer) antibodies were stained with Thermo Fisher Scientific’s Lab Vision^TM^DAB Plus substrate staining system (Fremont, CA, USA). Photographs were captured with a light microscope (Nikon, Tokyo, Japan).

### 2.11 Immunofluorescence (IF) staining

After being immobilized for 40 min in 4% paraformaldehyde solution, the MPMs and chondrocytes were penetrated for 15 min with 0.1% Triton-100X. Frozen sections were fixed with a mixture of acetone and methanol (1:1 ratio). Following 1 h of blocking with 10% goat serum, they were treated with the matching primary (anti-ASC for macrophages and anti-HMGB1 for chondrocytes) and secondary (Alexa 488 Goat Anti −Rabbit IgG antibody) antibodies, mounted using a mounting solution that contained 4′,6-diamidino-2-phenylindole (DAPI, Abcam, Cambridge, MA, USA), and captured on fluorescence microscopy (Nikon, Tokyo, Japan) images.

### 2.12 Lyso-Tracker Red staining

MPMs and chondrocytes seeded in glass bottom dishes (1 × 10^4^ per well) were incubated with pre-warmed Lyso-Tracker Red working solution (1 mL per well, Beyotime Biotechnology, Shanghai, China) for 30 min. After aspiration of the working solution, a fluorescent microscope (Nikon, Tokyo, Japan) was used to obtain pictures after adding 1 mL of fresh media.

### 2.13 Measurement of phagocytic activity

Macrophage phagocytosis was labeled with a pHrodo^TM^ Green *E. coli* BioParticles^TM^ conjugate (P35366, Invitrogen, Waltham, MA, USA). MPMs seeded in glass bottom dishes (1 × 10^4^ per well) were pretreated with Par (0.05, 0.25, and 1.25 μmol/L) and colchicine (10 nmol/L) for 1 h, and then stimulated with LPS (1 μg/mL) plus MSU (200 μg/mL) for 18 h. And then, pre-warmed promote Green working solution (100 μg/mL) was added (1 mL per well) for 1 h. A fluorescent microscope (Nikon, Tokyo, Japan) was used to obtain pictures.

### 2.14 Molecular docking

The molecular docking of NLRP3 of pyrin domain (PYD) and NAIP, CⅡTA, HET-E, and TP1 (NACHT) domain to Par was performed using AutoDock 4.2.6. The crystal structures of NLRP3 PYD domain (PDB ID: 3QF2) and NACHT domain (PDB ID: 8WSM) were obtained from Protein Data Bank (PDB, https://www.rcsb.org/). The 3D structure sdf file of Par (PubChem CID: 7251185) was downloaded from the PubChem database (https://pubchem.ncbi.nlm.nih.gov/). The AutoDock Tools version 1.5.6 package was used to generate the docking input files and analyze the docking results.

### 2.15 Statistical analysis

All data were evaluated mean ± standard deviation (SD) using GraphPad Prism 7.0 (GraphPad Software, Inc., San Diego, CA USA). One-way ANOVA and Tukey’s multiple comparison tests were used to determine statistical significance. *P* < 0.05 indicates a statistically significant difference between the groups.

## 3 Result

### 3.1 Par attenuated proinflammatory mediators in macrophages stimulated by MSU plus LPS

Par, with a molecular formula of C_15_H_20_O_3_, is a natural product of sesquiterpene lactones, and its *α*-methylene-*γ*-lactone structure is the basis of its pharmacological effects (Fig. 1A). To preliminarily explore the safety of Par, cell viability assay was conducted on MPMs, showing that Par didn’t affect cell viability of MPMs within 0 − 2.5 μmol/L, and 5 μmol/L Par exerted a slight cell toxicity (Fig. 1B). Therefore, concentrations of 0.05, 0.25, and 1.25 μmol/L of Par were chosen to investigate the therapeutic effect of Par on inflammation induced by LPS (1 μg/mL) and MSU (500 μg/mL) on macrophages. The secretion of IL-1*β* in the cell supernatant was detected by ELISA (Fig. 1C) and Western blotting (Fig. 1D). These results indicated that Par does-dependently decreased the level of IL-1*β* stimulated by LPS plus MSU. Besides, the expression of Cathepsin B and HMGB1 in the supernatant was detected by western blotting (Fig. 1D) as well, which further exhibited the effect of Par on depression of Cathepsin B and HMGB1. In addition, this study further detected the expression of NLRP3 inflammasome activation-related protein (NLRP3, ASC, Caspase-1, cathepsin B, and IL-1*β*) in MPMs (Fig. 1E and G), as well as the levels of IL-1*β*, Cathepsin B, and HMGB1 released in the supernatant (Fig. 1D and F) by Western blotting. Par and colchicine pretreatment significantly suppressed the expression of those inflammatory factors stimulated by MSU plus LPS. Furthermore, IF staining (Fig. 1H) was performed to intuitively observe the expression of ASC in MSU/LPS-induced MPMs and compare the therapeutic effects of Par and colchicine. As shown in Fig. 1H, Par dose-dependent reversed MSU/LPS-induced ASC upregulation. These results indicated that Par attenuated the inflammatory response in MSU-stimulated MPMs by inhibiting the activation and assemblage of NLRP3 inflammasomes.

### 3.2 Par attenuated proinflammatory mediators in chondrocytes stimulated by MSU plus LPS

This study further explored the impact of Par on chondrocytes stimulated by MSU in combination with LPS. Notably Par didn’t display any cytotoxicity at concentrations up to 5 μmol/L (Fig. 2A). Western blotting results presented that the expression of NLRP3, Caspase-1, Cathepsin B, and IL-1*β* was significantly increased induced by MSU (200 μg/mL) plus LPS (μg/mL), while Par pretreatment dose-dependently inhibited the expression of these proteins and 1.25 μmol/L Par demonstrated therapeutic effects generally superior to colchicine (Fig. 2B and C). Furthermore, IF staining results directly showed that Par also reduced the expression of HMGB1 in chondrocytes in a dose-dependently manner, thereby inhibiting the activation of NLRP3 inflammasome (Fig. 2D). The aforementioned results indicated that Par alleviated the inflammatory response in MSU/LPS-stimulated chondrocytes by diminishing the expression of proteins associated with NLRP3 inflammasome assembly and eventually suppressing the activation of NLRP3 inflammasome.

**Fig. 2:**
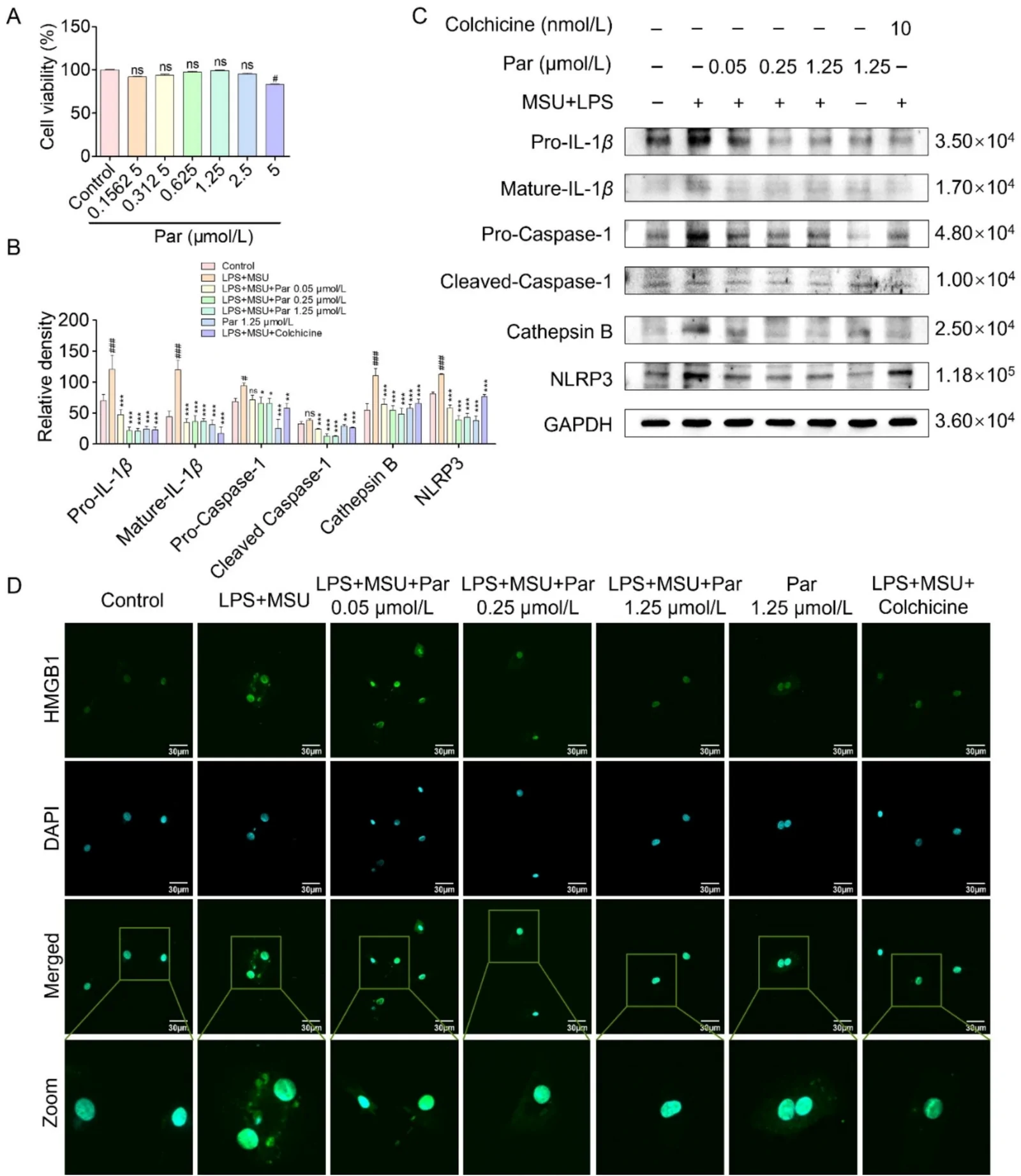
Par attenuated proinflammatory mediators in chondrocytes stimulated by MSU plus LPS. (A) Cell viability of chondrocytes was determined by CCK-8 assay. (B) Each immunoreactive band of NLRP3, Caspase-1, Cathepsin B, and IL-1*β* was normalized against GAPDH. (C) Expression of NLRP3, Caspase-1, Cathepsin B, and IL-1*β* in chondrocytes detected by Western blotting. (D) Immunofluorescence staining of HMGB1 (× 600, scale bar 30 μm). Mean ± SD, *n* = 3. ^#^*P* < 0.05, ^###^*P* < 0.001 *vs* control group; *P < 0.05, ^**^P < 0.01, ^***^P < 0.001 *vs* LPS + MSU group; ns indicates no significant difference. LPS: lipopolysaccharide; NLRP3: NOD-Like receptor pyrin domains 3; CCK8: cell counting kit-8; GAPDH: glyceraldehyde-3-phosphate dehydrogenase; MSU: monosodium urate; HMGB1: high mobility group box-1.

### 3.3 Par ameliorated inflammation response by promoting phagocytic capacity of macrophage and chondrocyte

This study used pHrodo^TM^ Green and Lyso-Tracker Red lysosomal fluorescent probes to double-stain macrophages and chondrocytes to explore whether the anti-inflammatory effects of Par could promote phagocytosis. PHrodo^TM^ Green is a novel fluorescent probe for measuring intracellular pH in living cells that emits bright green fluorescence in acidic pH environments such as phagosomes. Lyso-Tracker Red is an eosinophilic fluorescent probe used to label living cell lysosomes, selectively retained in acidic lysosomes. IF staining results showed that in MSU plus LPS-induced MPMs, the fluorescence intensity of pHrodo^TM^ Green and Lyso-Tracker Red was significantly reduced, accompanied by a reduction in the quantity of phagosomes and lysosomes. In contrast, pretreatment with Par led to an augmentation in the number of phagosomes and lysosomes, indicating a progressive restoration of macrophage phagocytosis (Fig. 3A). Likewise, a significant enhancement in fluorescence intensity was observed in chondrocytes of MSU/LPS-stimulated Par group. Moreover, the number of phagolysosomes and lysosomes exhibited a gradual increment with increasing concentrations of Par, indicating that Par facilitated the restoration of the phagocytic capacity of chondrocytes (Fig. 3B). Above results indicated that Par ameliorated MSU/LPS-induced inflammation response by inhibiting the reduction of phagosomes and lysosomes and promoting macrophage and chondrocyte phagocytosis capacity.

**Fig. 3:**
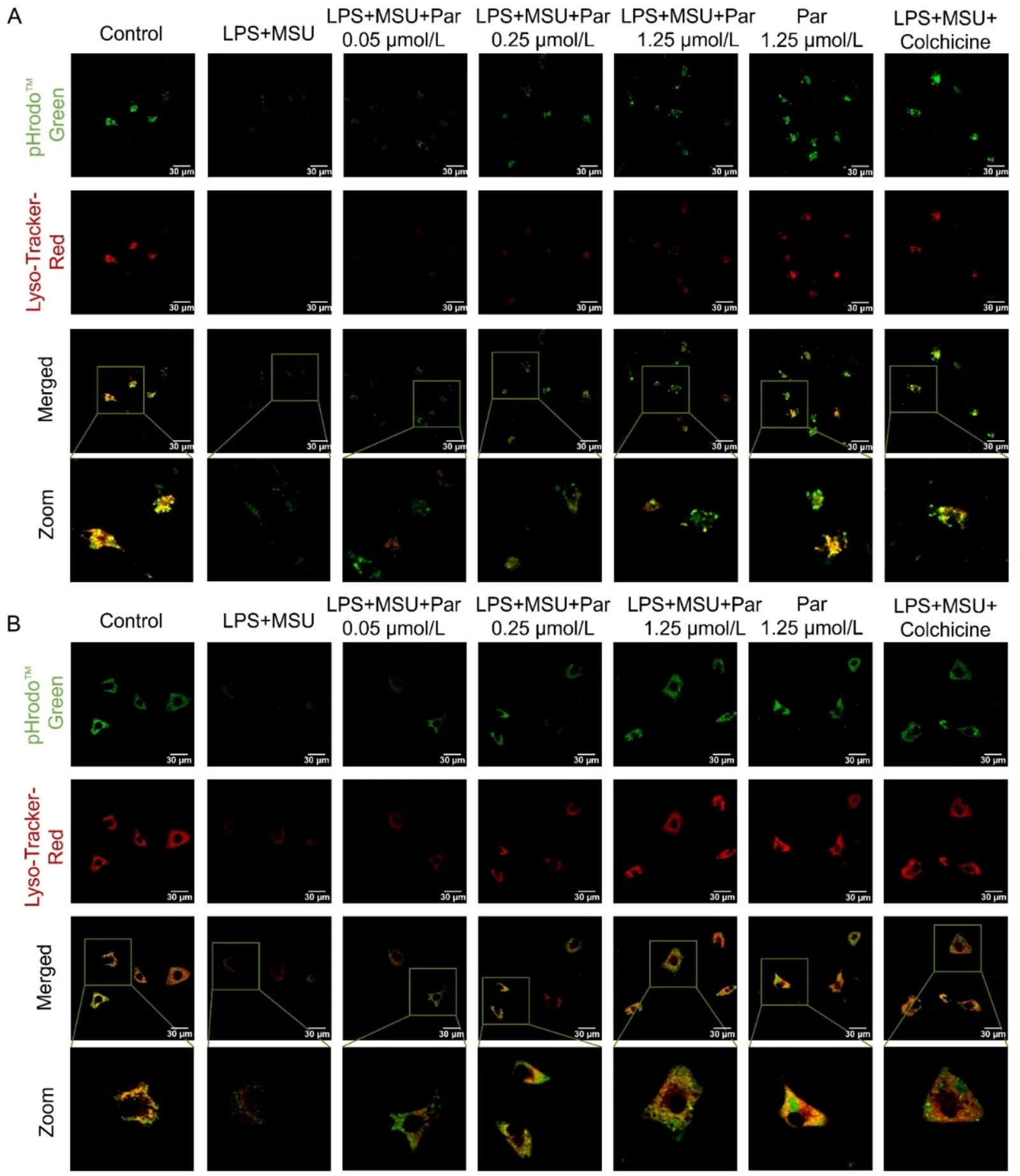
Par ameliorated inflammation response by promoting macrophage and chondrocyte phagocytosis ability. (A) pHrodo^TM^ Green assay for MPMs phagocytosis (green fluorescence, × 600, scale bar 30 μm) and Lyso-Tracker Red lysosomal fluorescent probe labeling staining (red fluorescence, × 600, scale bar 30 μm). (B) pHrodo^TM^ Green assay for chondrocyte phagocytosis (green fluorescence, × 600, scale bar 30 μm) and Lyso-Tracker Red lysosomal fluorescent probe labeling staining (red fluorescence, × 600, scale bar 30 μm). LPS: lipopolysaccharide; MSU: monosodium urate; MPM: mouse peritoneal macrophage.

### 3.4 Par ameliorated renal impairment in hyperuricemic mice

The therapeutic effect of Par on hyperuricemia was investigated, which is closely associated with gout, by establishing the hyperuricemia model induced by OXO in combination with YE (Fig. 4A) and subsequently detecting the serum levels of UA, XOD, CRE, and BUN. The biochemical assays indicated that, in comparison with the OXO + YE group, serum levels of UA, XOD, CRE, and BUN were dose-dependently decreased with Par (Fig. 4B − E). In addition, H&E staining of pathological sections of the kidney showed that the renal tubular in the OXO + YE group was scattered, the glomeruli were mildly swollen, and the glomerular walls were significantly thinner. Besides, a large amount of cell nuclei aggregated in the periglomerular region of OXO + YE group, indicating the presence of inflammatory infiltration. However, Par effectively ameliorated OXO + YE-induced the renal injury by promoting the recovery of glomerular morphology, improving nuclear distribution, and reducing inflammatory infiltration of the periglomerular region (Fig. 4F). In summary, Par effectively alleviated the renal impairment in hyperuricemic mice.

**Fig. 4:**
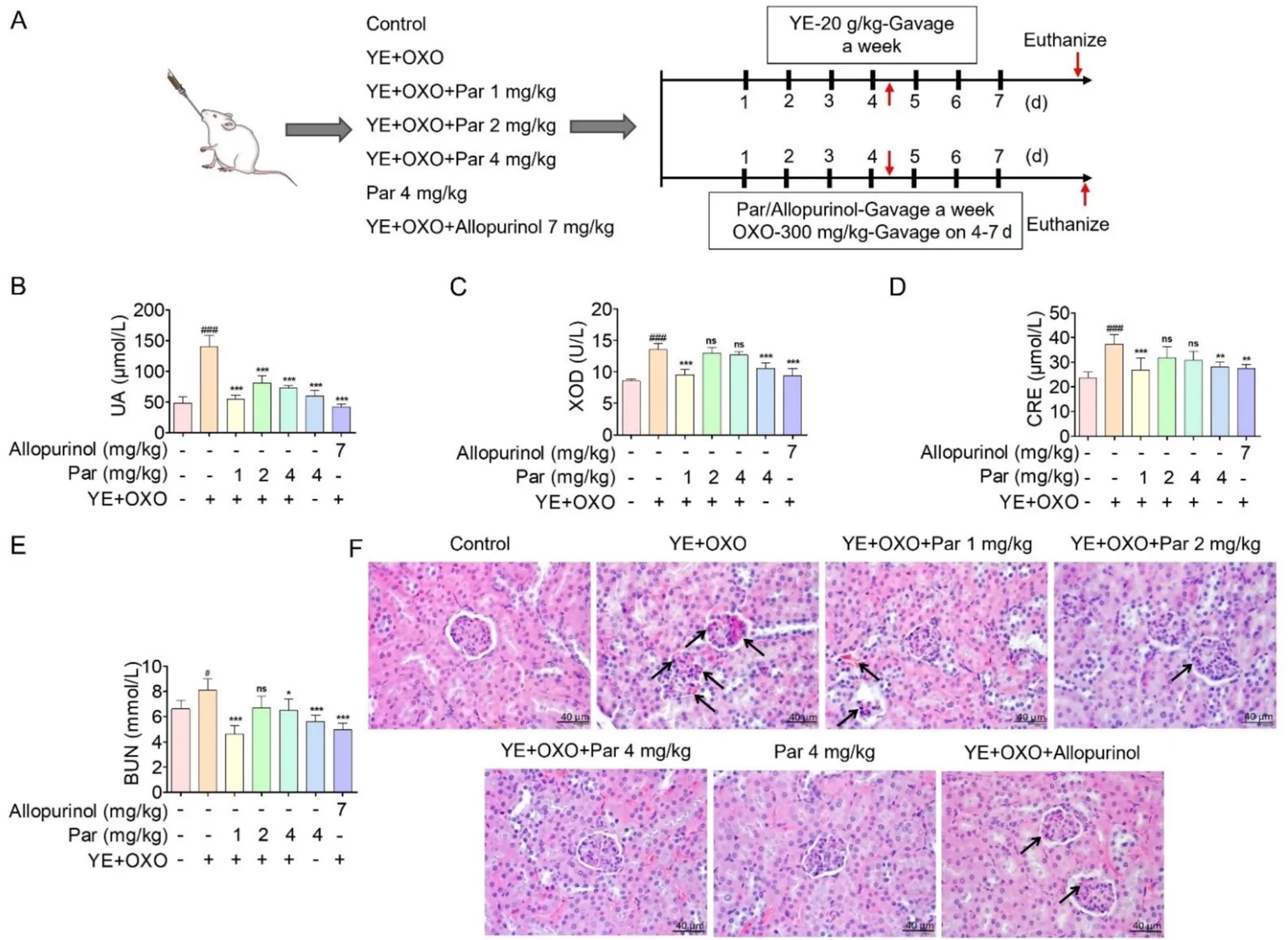
Par ameliorated renal injury in mice with hyperuricemia. (A) Establishment of mouse hyperuricemia model by OXO plus YE induction (five mice per group and representative images were shown for histopathological changes). (B − E) Serum uric acid (UA), xanthine oxidase (XOD), creatinine (CRE), and blood urea nitrogen (BUN) levels. (F) HE staining (× 400, scale bar 40 μm) of the kidney (black arrows point the scattered renal tubular, mildly swollen glomeruli, thinning glomerular walls, and aggregated cell nuclei). Mean ± SD, *n* = 3. ^#^*P* < 0.05, ^###^*P* < 0.001 *vs* control group; *P < 0.05, ^***^P < 0.001 *vs* YE + OXO group; ns indicates no significant difference. OXO: oxygen oxazine; YE: yeast extract; HE: hematoxylin and eosin.

### 3.5 Par reduced inflammatory infiltration in mouse gouty arthritis model

Considering the efficacy and safety of Par, this study selected 1, 2, and 4 mg/kg doses to establish an acute gouty arthritis model (Fig. 5A) based on the *in vivo* dose (1 − 10 mg/kg) in previous efficacy studies of Par (Zhan et al., 2024). The histomorphology records and paw thickness data of mouse paws showed that Par significantly reduced the degree of MSU-induced paw redness and swelling (Fig. 5B and C). HE staining results indicated that nuclei aggregates were visible in the muscle layer of the paw of mice in the MSU group, which was ameliorated by Par (Fig. 5D). As shown in immunohistochemical staining (Fig. 5E and F), the expression of lymphocyte antigen 6 complex (Ly6G) and EGF-like module-containing mucin-like hormone receptor 1 (F4/80), markers of neutrophils and macrophages respectively, in paw tissues was significantly increased after MSU crystal stimulation, indicating that macrophages and neutrophils were massively recruited in the paw tissues, which were significantly reduced with Par treatment. These results demonstrated that Par could reduce inflammatory infiltration in mouse gouty arthritis model.

**Fig. 5:**
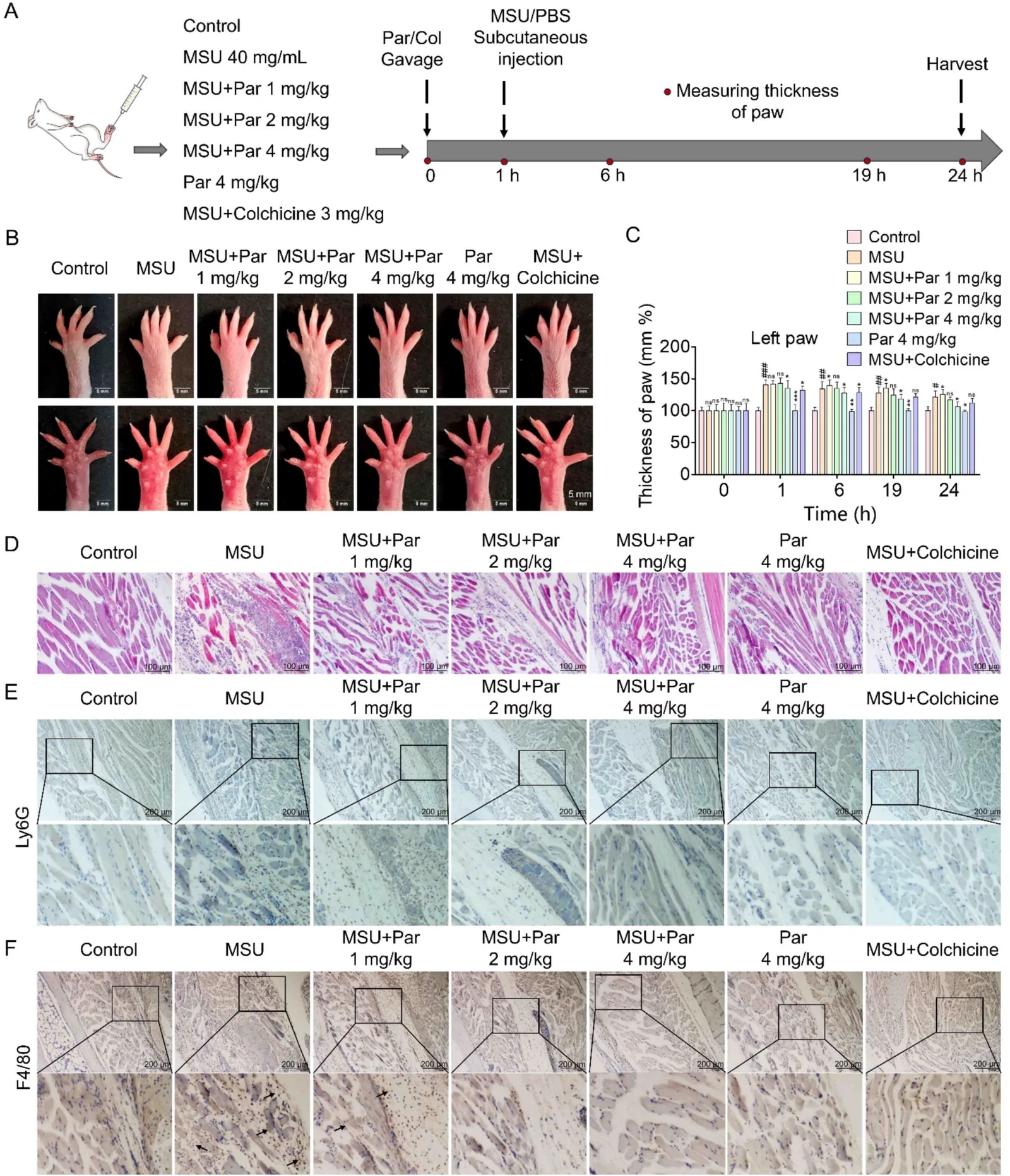
Par reduced inflammatory infiltration in murine gouty arthritis. (A) Gouty arthritis model established by MSU injection (five mice per group and representative images were shown for histopathological changes). (B) Histomorphology of mice paws (×2, scale bar 5 mm). (C) Changes in paw thickness after 0, 1, 6, 19, and 24 h of MSU crystal treatment. (D) HE staining (× 200, scale bar 100 μm) of paw tissue. (E and F) Immunohistochemical staining of paws tissue pathology sections (E) Ly6G (× 100, scale bar 200 μm) and (F) F4/80 (× 100, scale bar 200 μm). Mean ± SD, *n* = 3. ^#^*P* < 0.05, ^##^*P* < 0.01, ^###^*P* < 0.001 *vs* control group; *P < 0.05, ^***^P < 0.001 *vs* MSU group; ns indicates no significant difference. PBS: phosphate-buffered saline; MSU: monosodium urate; Ly6G: lymphocyte antigen 6 complex; F4/80: EGF-like module-containing mucin-like hormone receptor-like 1.

### 3.6 Par attenuated release of inflammatory mediators in gouty arthritis model by inhibiting NLRP3 inflammasome

To investigate the therapeutic mechanism of Par on gouty arthritis, the expression of NLRP3 inflammasome relevant proteins was detected by Western blotting. As shown in Fig. 6A and C, the levels of NLRP3, ASC, Caspase-1, and IL-1*β* were significantly upregulated by MSU stimulation and dose-dependently reduced with Par treatment, indicating that Par inhibited the assembly and activation of NLRP3 inflammasomes, reduced the maturation and release of IL-1*β*, thereby preventing further expansion of inflammation. Additionally, phagocytosis of MSU crystals by macrophages into lysosomes results in lysosomal damage, which in turn leads to an augmented release of Cathepsin B. HMGB1, a chromosome-binding protein abundantly present in eukaryotic cells, is released into the extracellular compartment to produce a wide range of biological effects following cell injury or necrosis. The protease known as neutrophil elastase (NE) is secreted by neutrophils and is involved in the control of inflammation and immunological response. The protein expression of Cathepsin B, HMGB1, and NE further was examined using Western blotting analysis. In contrast to the MSU-treated group, the expression of these proteins was reduced in a dose-dependent manner upon treatment with Par. (Fig. 6B and D). This finding implies that Par alleviated the MSU-induced impairment of macrophage phagocytic ability by inhibiting lysosome damage. Moreover, Par restrained the amplification of the inflammatory response and the recruitment of neutrophils by curtailing the release of HMGB1. Moreover, the immunohistochemistry findings provided an intuitive visualization of NLRP3 in the tissue (Fig. 6E), consistent with the outcomes obtained from Western blotting analysis that Par effectively repressed the NLRP3 expression of paw tissue. In conclusion, these results indicated that Par attenuated the release of inflammatory mediators in gouty arthritis by inhibiting the NLRP3 inflammasome.

**Fig. 6:**
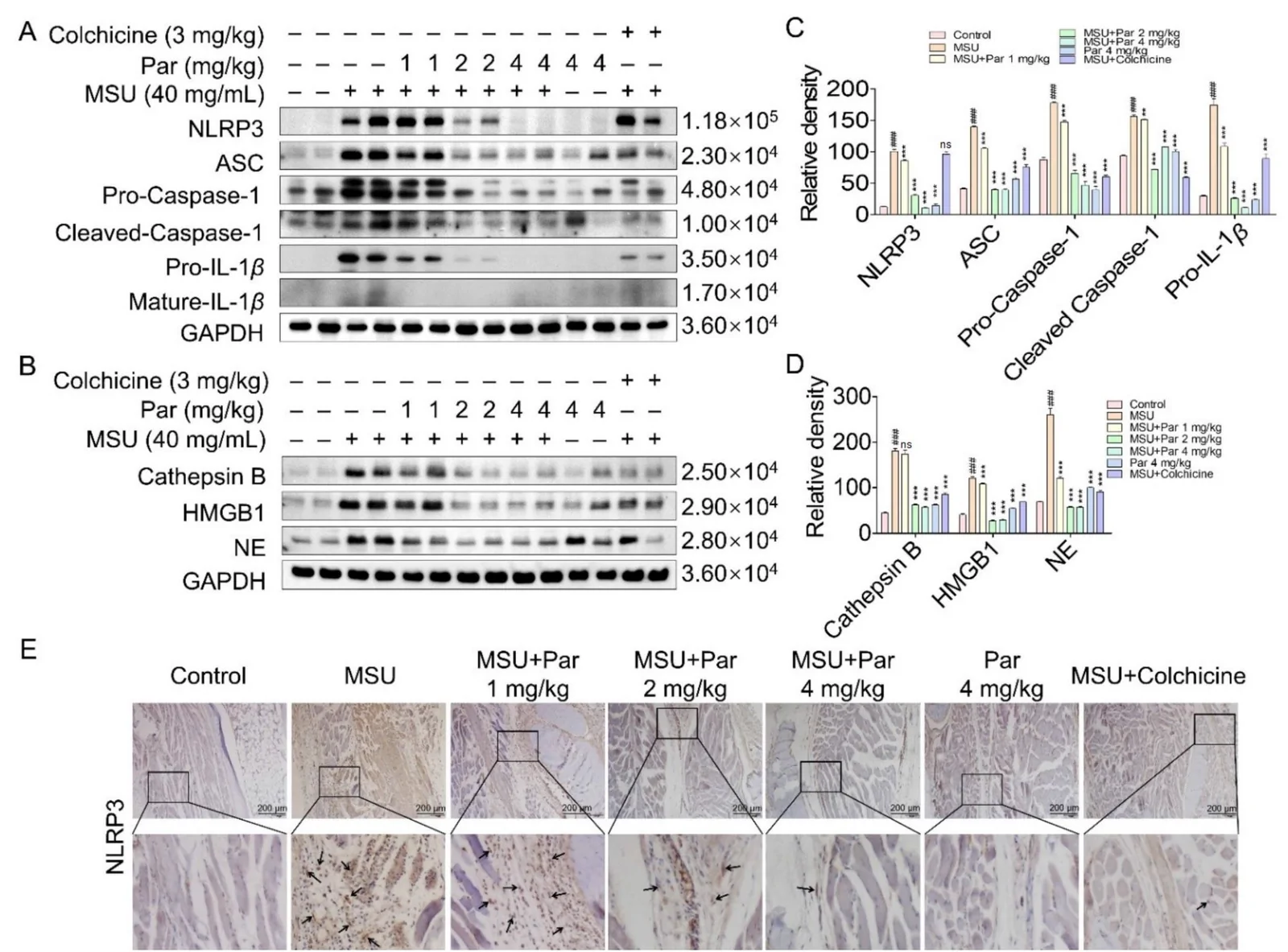
Par attenuated release of inflammatory mediators in gouty arthritis by inhibiting NLRP3 inflammasome. (A) Expression of NLRP3, ASC, Caspase-1, and IL-1*β* in paws detected by Western blotting. (B) Expression of Cathepsin B, NE, and HMGB1 in paws by Western blotting. (C and D) Each immunoreaction band was normalized against GAPDH. (E) Immunohistochemistry for NLRP3 (× 100, scale bar 200 μm). Mean ± SD, *n* = 3. ^###^*P* < 0.001 *vs* control group; ^***^P < 0.001 *vs* MSU group; ns indicates no significant difference. MSU: monosodium urate; NLRP3: NOD-like receptor pyrin domains 3; NE: neutrophil elastase; ASC: apoptosis-associated speck-like protein containing a CARD; GAPDH: glyceraldehyde-3-phosphate dehydrogenase.

### 3.7 Par reduced inflammatory infiltration in mouse air pouch gout model

To investigate the effect of Par on inflammatory synovial lesions of the gouty joint, this study established the air pouch gout model (Fig. 7A) (Fehrenbacher & McCarson, 2021). H&E staining demonstrated that a significant amount of nuclear aggregation was detected subsequent to the injection of MSU crystals, while this aggregation was attenuated following the administration of Par and colchicine (Fig. 7B). Immunohistochemical and immunofluorescence staining were used to detect the infiltration of inflammatory cells. Around the control group, no discernible brown-yellow Ly6G or F4/80 positive region was observed (Fig. 7C and D). As the administration concentration increased, there was a substantial rise in the positive staining area of Ly6G in the MSU group and a decrease in the positive expression of Ly6G in the Par-administered group (Fig. 7C). The fluorescence intensity of F4/80 in subcutaneous tissues of mice was enhanced after MSU stimulation, while it was weakened in the Par and colchicine-treated groups (Fig. 7D). Notably, the degree of reduction of fluorescence intensity of F4/80 in the high-dose Par group was nearly equivalent to that of the control group. The above results indicated that Par ameliorated MSU crystal-induced the infiltration of inflammatory cell within the simulated gout-like inflammatory synovial lesions.

**Fig. 7:**
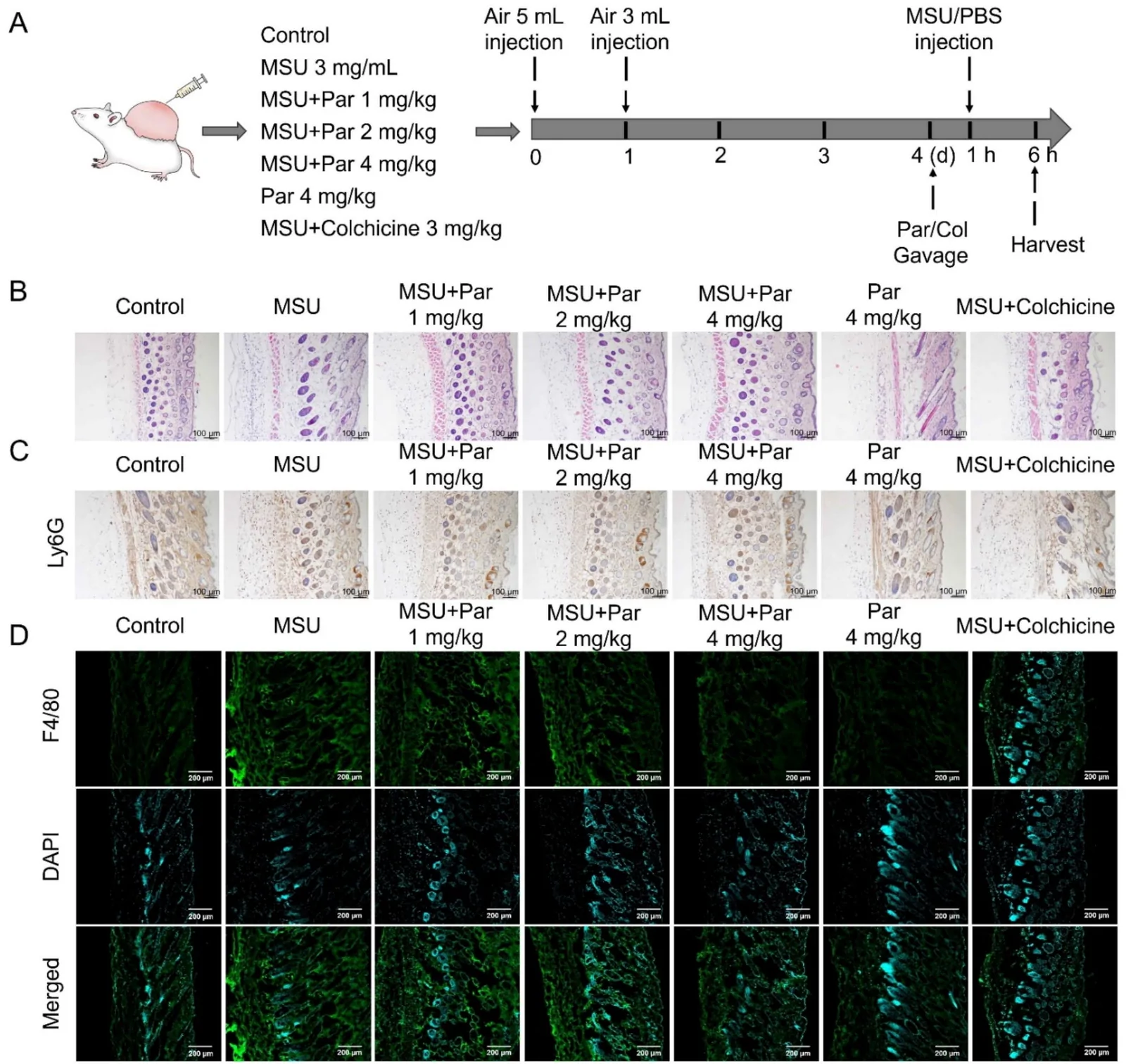
Par reduced inflammatory infiltration in mouse air pouch gout model. (A) Air pouch gout model established by MSU injection (five mice per group and representative images were shown for histopathological changes). (B) HE staining of (× 100, scale bar 100 μm) skin tissue. (C) Immunohistochemical staining for Ly6G (× 100, scale bar 100 μm). (D) Immunofluorescence staining for F4/80 (× 100, scale bar 200 μm). MSU: monosodium urate; PBS: phosphate-buffered saline; Ly6G: lymphocyte antigen 6 complex; F4/80: mouse EGF-like module-containing mucin-like hormone receptor-like 1; DAPI: 4′,6-diamidino-2-phenylindole.

### 3.8 Par inhibited activation of NLRP3 inflammasome and decreased production of inflammatory mediators in air pouch gout model

To investigate the therapeutic mechanism of Par on the air pouch gout model, the expression of NLRP3 inflammasome relevant proteins was detected. Western blotting results showed that the expression of NLRP3, ASC, Caspase-1, and IL-1*β* was reduced in Par and colchicine groups, with the most pronounced reduction in the Par high-dose group (Fig. 8A and C), indicating that Par inhibited the activation of the NLRP3 inflammasome, thereby reducing inflammation in the air pouch gout model. Similarly, Par considerably reduced the levels of Cathepsin B, HMGB1, and NE caused by MSU in mice’s subcutaneous tissue, alleviating the inflammatory response (Fig. 8B and D). Additionally, the fluorescence intensity of NLRP3 was dose-dependently decreased with Par (Fig. 8E). These results indicated that Par inhibited the activation of the NLRP3 inflammasome and decreased the production of inflammatory mediators in the air pouch gout model.

**Fig. 8:**
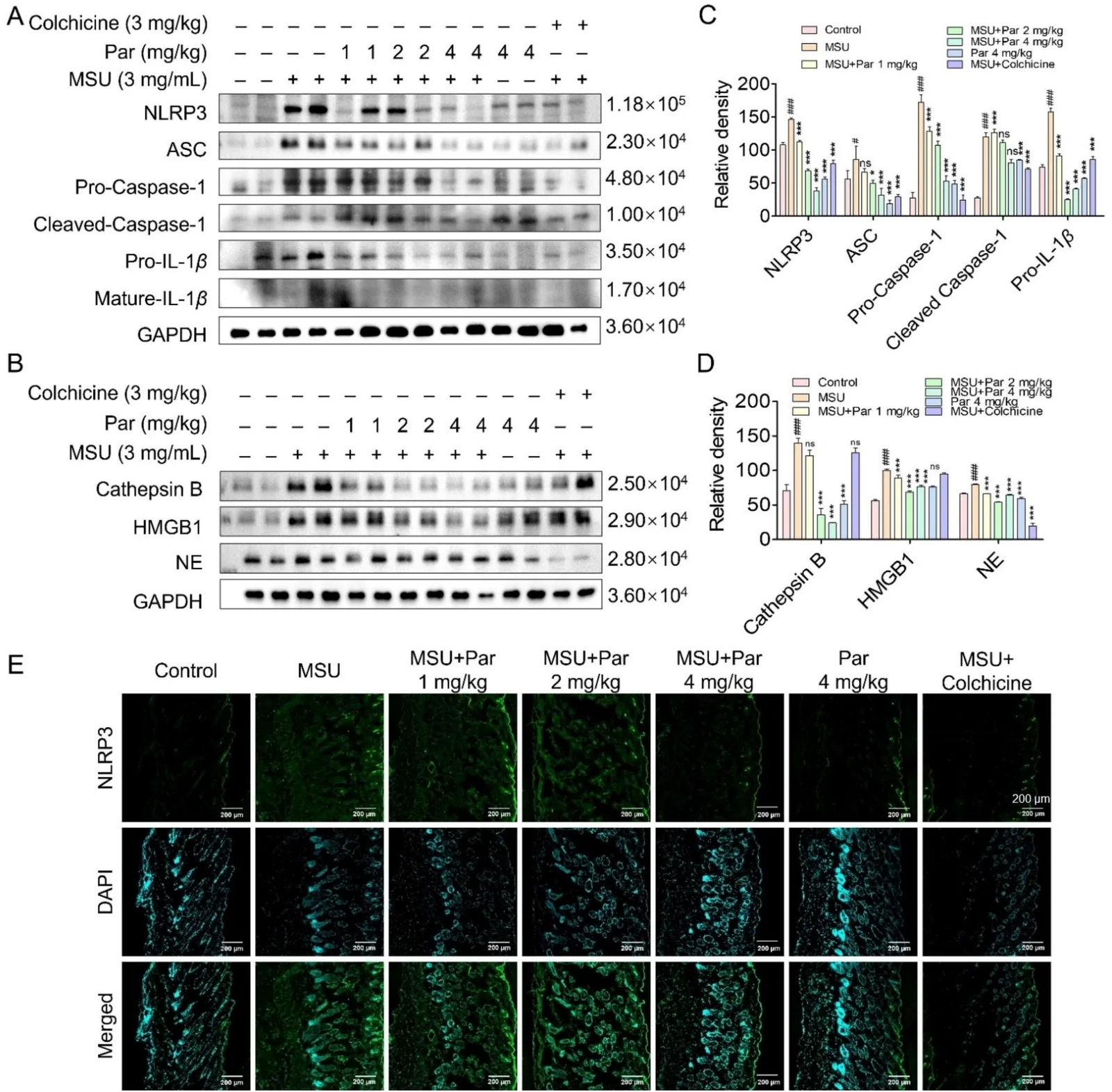
Par inhibited NLRP3 inflammasome’s activation and decreased production of inflammatory mediators in air pouch gout model. (A) Expression of NLRP3, ASC, Caspase-1, and IL-1*β* in back skin detected by Western blotting. (B) Expression of Cathepsin B, NE, and HMGB1 in back skin by Western blotting. (C and D) Each immunoreaction band was normalized against GAPDH. (E) Immunofluorescence for NLRP3 (× 100, scale bar 200 μm). Mean ± SD, *n* = 3. ^#^*P* < 0.05, ^###^*P* < 0.001 *vs* control group; ^***^P < 0.001 *vs* MSU group; ns indicates no significant difference. ASC: apoptosis-associated speck-like protein containing a CARD; DAPI: 4′,6-diamidino-2-phenylindole; GAPDH: glyceraldehyde-3-phosphate dehydrogenase; HMGB1: high mobility group box-1; NE: neutrophil elastase; NLRP3: NOD-like receptor pyrin domains 3; MSU: monosodium urate.

### 3.9 Molecular docking analysis of potential binding of Par to NLRP3 binding site

This study constructed a molecular docking model to elucidate the interaction mechanism between Par and the PYD and NACHT domains of NLRP3. The results proved that Par could be docked into the PYD domain (Fig. 9A). The binding model of PYD and Par, as also determined through theoretical chemistry, disclosed that the chain of Par was able to form strong interactions with ARG-43 and GLY-44 by theoretical chemistry using the same method (Fig. 9B). As shown in Fig. 9C, Par could be docked into the NACHT domain as well. In addition, the key interactions between Par and NACHT domain was hydrogen bond interaction with ARG-578 (Fig. 9D). These findings imply that Par has the potential to specifically target the active domain of NLRP3.

**Fig. 9:**
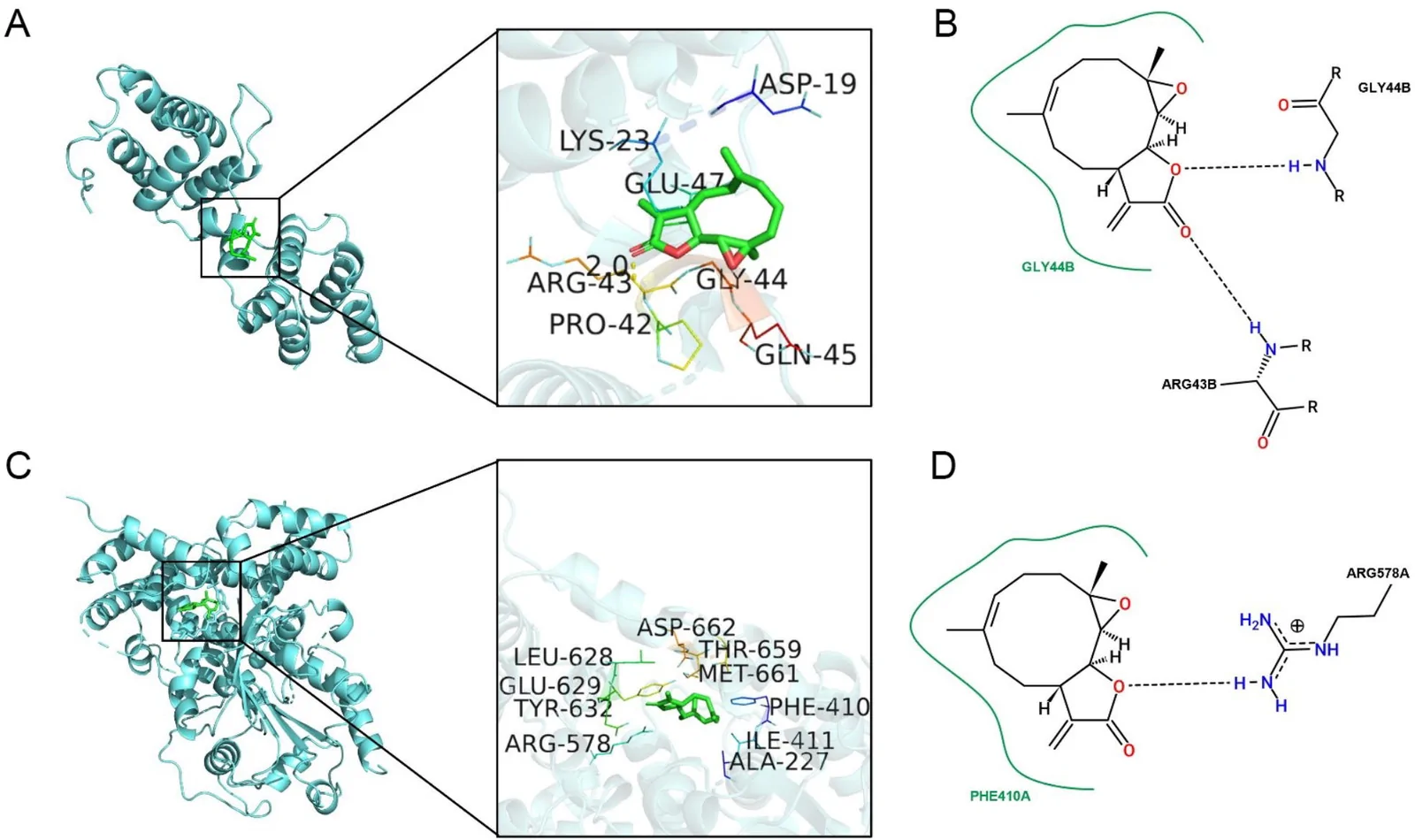
Binding mode of Par between PYD and NACHT domains of NLRP3 protein. (A) Schematic representations 3D of the binding interactions between the PYD active site and Par. (B) Schematic representations 2D of the binding interactions: hydrogen bond interaction between the PYD active site and Par. (C) Schematic representations 3D of the binding interactions between the NACHT active site and Par. (D) Schematic representations 2D of the binding interactions: hydrogen bond interaction between the NACHT active site and Par. PYD: pyrin domain; ASP: aspartic acid; LYS: lysine; GLU: glutamic acid; ARG: arginine; GLY: glycine; PRO: proline; GLN: glutamine; LEU: leucine; THR: threonine; MET: methionine; TYR: tyrosine; PHE: phenylalanine; ILE: isoleucine; ALA: alanine.

## 4 Discussion

The burden of gout is increasing worldwide due to the increasing prevalence of gout-related comorbidities and all-cause mortality (Yuliya, Danve, & Neogi, 2022). However, colchicine and corticosteroids, which are commonly used in clinical practice for the prevention and treatment of gouty arthritis, have toxic side effects such as gastrointestinal bleeding, hepatotoxicity and nephrotoxicity (Angelidis et al., 2018). Therefore, it is necessary to explore a new, safe, low-toxicity and effective anti-gout drug. Par, a sesquiterpene lactone, has been demonstrated to possess favorable anti-inflammatory and redox modulatory capabilities (Junan et al., 2020; Pavan, Suhas, Dhananjay, Ajay, & Mukesh, 2022). However, the effects of Par on gouty arthritis remain unexamined. Therefore, this study explored the potential impacts of Par in gouty arthritis, as well as unraveled its underlying molecular mechanism.

To preliminarily explore the therapeutic effect of Par on MSU induced gouty inflammation, this study established two *in vitro* models stimulated by LPS/MSU: macrophages, which are innate immune cells present within the joints; chondrocytes, representing the sole cellular component on the surface of articular cartilage tissue. When MSU is recognized and engulfed by macrophages. Subsequently, downstream inflammatory factors are released accompanied with pyroptosis, which then recruit neutrophils to propagate the inflammation, ultimately culminating in acute gout flares (Nicola, Anna, Angelo, & Abhishek, 2021). In addition, the construction and activation of NLRP3 inflammasomes also depend on Cathepsin B (Peng et al., 2022). The close regulation between HMGB1 and NLRP3 inflammasomes stimulates the onset of various inflammatory diseases (Choe, Choi, Park, & Kim, 2018). Chondrocyte components mainly include proteoglycans and collagen, which maintain the homeostasis of the internal environment of articular cartilage tissues and the death of the chondrocytes heralds the degenerative pathological changes in articular cartilage tissues (Xu et al., 2023). The Western blotting and immunohistochemical results showed that Par significantly decreased LPS/MSU-induced inflammatory response by reducing the expression of NLRP3 inflammasome activation and assembly related proteins (ASC, Caspase-1, and NLRP3) in macrophages and chondrocytes. Moreover, Par reduced the levels of inflammatory factor IL-1*β*, which is downstream of the NLRP3 signaling pathway, both within intracellular and extracellular. These findings suggest that Par might inhibit LPS/MSU-induced inflammatory response by suppressing the activation and assembly of NLRP3 inflammasomes and depressing the maturation and release of downstream IL-1*β*, thereby potentially preventing the further dissemination of inflammation (Fig. 1, Fig. 2).

Phagocytosis is an essential function of acute inflammation. MSU crystals are phagocytosed by macrophages and chondrocytes, causing lysosomal disruption and release of lysosomal proteases and histones, which act on the NLRP3 inflammasome to trigger its activation (So and Martinon, 2017, Zhao et al., 2022). The phagocytic activity of MPMs and chondrocytes was significantly reduced by LPS/MSU stimulation, while Par restored their normal phagocytic activity dose-dependently (Fig. 3), indicating that Par inhibited the reduction of phagosomes and lysosomes and promoted macrophage and chondrocyte phagocytosis ability to ameliorate MSU/LPS-induced inflammation response.

Elevated uric acid levels are undoubtedly the main risk factor for gout flares and a direct result of hyperuricemia. Serum urate excretion is regulated by urate transporters in the kidneys, however persistent overload of uric acid metabolism and urate deposition in the kidneys would lead to hyperuricemia nephropathy (Vargas-Santos, & Neogi, 2017). Hyperuricemia and gout do not necessarily occur together, but their coexistence will certainly further aggravate each other's condition. Uric acid lowering therapy is one of the most effective long-term treatments for gout (Nicola, Anna, Angelo, & Abhishek, 2021). Therefore, drugs that can simultaneously treat gout and hyperuricemia are undoubtedly superior to other anti-gout drugs. This study established a hyperuricemia model to explore the anti-gout potential of Par, and found that Par dose-dependently reduced serum uric acid levels, and alleviated kidney damage caused by reducing XOD, BUN, CRE levels, and renal pathological changes in the hyperuricemia model (Fig. 4), indicating that Par has demonstrated great potential in gout treatment through its protective effect on the kidneys.

To further confirm the anti-inflammatory effect of Par on gout and investigate the *in-vivo* effect of Par on inflammatory synovial lesions of the gouty joint, this study established the acute gouty arthritis model by injecting MSU crystals into the mice paw and the air pouch gout model. It was recorded that the *in vivo* dosages studied were 250 and 500 mg/kg in early pharmacological studies of Par (Zingarelli, Hake, Denenberg, & Wong, 2002). Moreover, a calibration curve for a linear regression concentration range of 2 − 500 ng/mL was established through pharmacokinetic studies of Par (the concentration of Par in rat plasma after intravenous injection of a single dose of 5 mg/kg Par for 1 h was 57.33 ng/mL) (Zhao, Zhao, Zhang, Pan, & Huo, 2016). Therefore, this study chose Par doses of 1, 2, and 4 mg/kg for *in vivo* experiments, considering the efficacy and safety of *in vivo* administration. The morphological and histopathological examination results of the paw showed that Par significantly inhibited the expression of NLRP3, ASC, Caspase-1, and IL-1*β* in the NLRP3 inflammasome signaling pathway while reducing the protein expression of inflammatory mediators Cathepsin B, HMGB1, and NE, indicating that by preventing NLRP3 activation, Par was able to decrease the release of relevant inflammatory components and alleviate inflammatory responses triggered by MSU crystals in the gouty arthritis model and the air pouch gout model.

This study investigated the molecular mechanism of Par in gout-like inflammation treatment and identified the target of Par through molecular docking. Par inhibited the activation and assembly of NLRP3 inflammasomes in macrophages and chondrocytes, preventing the recruitment of neutrophils and the expansion of inflammatory responses. However, aggregated neutrophils can dissolve gout inflammation by forming extracellular traps (Nicola, Anna, Angelo, & Abhishek, 2021). Our current study preliminarily detected the inhibitory effect of Par on neutrophil recruited by IL-1*β*, however, further researches are warranted to elucidate the specific impacts of Par on aggregated neutrophils.

## 5 Conclusion

Overall, Par has the potential to alleviate acute gout-like inflammation, which is achieved by impeding the activation of NLRP3 inflammasomes and curtailing the migration of neutrophils and macrophages, rejuvenating the phagocytic capabilities of MPMs and chondrocytes. Moreover, this research has proffered a novel therapeutic approach for gout treatment by Par through rectifying purine metabolism disorders instigated by high uric acid levels, and relieving the recurrence of gout flares (Fig. 10).

**Fig. 10:**
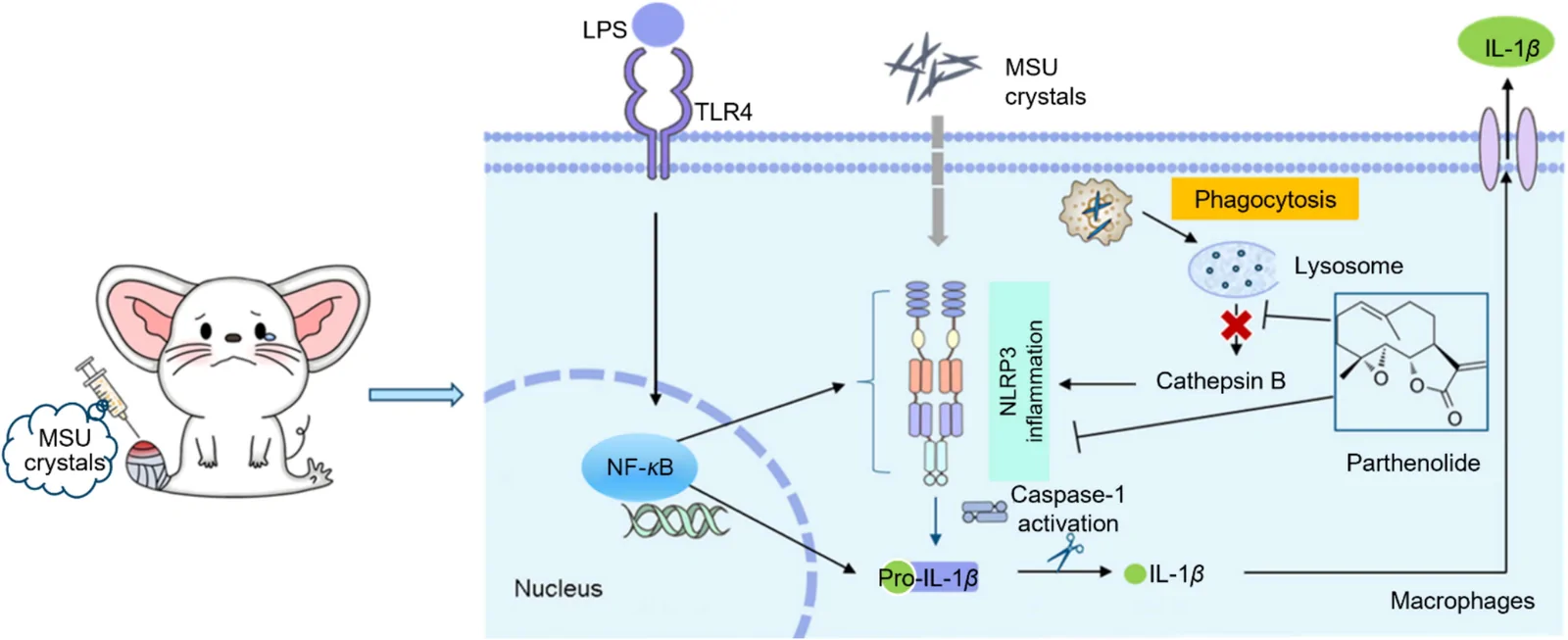
Par inhibited destruction of lysosomal membrane by engulfing MSU and activation of NLRP3 inflammasome stimulated by MSU, and prevented the spread of acute gouty like inflammation by reducing the maturation and release of inflammatory factor IL-1*β*. MSU: monosodium urate; NLRP3: NOD-like receptor pyrin domains 3; LPS: lipopolysaccharide; TLR: Toll-like receptor; NF-*κ*B: nuclear factor kappa-light-chain-enhancer of activated B cells.

## CRediT authorship contribution statement

**Yonghong Cheng:** Investigation, Formal analysis, Writing – original draft. **Yinjing Jiang:** Investigation, Formal analysis, Writing – original draft. **Hui Wang:** Investigation. **Haoqing Zhu:** Investigation, Formal analysis, Writing – original draft. **Yanling Wu:** Formal analysis, Writing – review & editing. **Jixing Nan:** Conceptualization, Funding acquisition, Supervision, Writing – review & editing. **Lihua Lian:** Conceptualization, Funding acquisition, Supervision, Writing – review & editing.

## Declaration of competing interest

The authors declare that they have no known competing financial interests or personal relationships that could have appeared to influence the work reported in this paper.
